# Relationship between Vancomycin Trough Serum Concentrations and Clinical Outcomes in Children: a Systematic Review and Meta-Analysis

**DOI:** 10.1128/aac.00138-22

**Published:** 2022-07-13

**Authors:** Lu Cao, Zhuo Li, Peng Zhang, Suyun Yong

**Affiliations:** a Department of Pharmacy, Shaanxi Provincial People's Hospital, Xi’an, China

**Keywords:** meta-analysis, vancomycin, therapeutic drug monitoring (TDM), trough concentrations, children

## Abstract

To systematically evaluate the relationships between vancomycin trough serum concentrations and clinical outcomes in children using meta-analysis. Several databases, including PubMed, Elsevier, Web of Science, EMBASE, Medline, clinicaltrials.gov, the Cochrane Library, and three Chinese databases (Wanfang Data, China National Knowledge Infrastructure, and SINOMED), were comprehensively searched to obtain research articles on vancomycin use in children from inception through December 2021. All studies were screened and evaluated using the Cochrane systematic review method. Then, the feature information was extracted for meta-analysis. The evaluated results included clinical efficacy, vancomycin-associated nephrotoxicity, hepatotoxicity, ototoxicity, mortality, and microbial clearance. A total of 35 studies involving 4820 children were included in the analysis. The meta-analysis showed that compared with children with vancomycin trough concentrations <10 μg/mL, those with vancomycin trough concentrations ≥10 μg/mL had a higher clinical efficacy rate [OR: 2.23, 95% CI: 1.29 to 3.84, *P* = 0.004] and higher incidences of nephrotoxicity [OR: 2.76, 95% CI: 1.51 to 5.07, *P* = 0.001], ototoxicity [OR: 1.87, 95% CI: 1.08 to 3.23, *P* = 0.02] and microbial clearance [OR: 2.36, 95% CI: 1.53 to 3.64, *P* = 0.0001]. All-cause mortality [OR: 1.07, 95% CI: 0.45 to 2.53, *P* = 0.88] and hepatotoxicity [OR: 0.84, 95% CI: 0.46 to 1.53, *P* = 0.57] were similar between the two groups. Subgroup analysis showed that compared with children with vancomycin trough concentrations of 10 to 15 μg/mL, those with vancomycin trough concentrations >15 μg/mL had a higher incidence of nephrotoxicity [OR: 2.64, 95% CI: 1.28 to 5.43, *P* = 0.008], but there was no significant difference in clinical efficacy [OR: 0.85, 95% CI: 0.30 to 2.44, *P* = 0.76]. A vancomycin trough concentration of 10 to 15 μg/mL can improve clinical efficacy in children. Additionally, avoidance of trough concentrations >15 μg/mL can reduce the incidence of adverse reactions.

## INTRODUCTION

Vancomycin is a glycopeptide antibiotic primarily used for Gram-positive infections, including methicillin-resistant Staphylococcus aureus (MRSA) and Enterococcus faecium infections. Vancomycin is a common cause of serious hepatotoxicity, nephrotoxicity, and ototoxicity and especially occurs in treatment with higher trough concentrations and longer durations of therapy (1, 2). According to the previous recommendations of the Infectious Diseases Society of America (IDSA), vancomycin serum trough concentrations ranging from 10 to 20 μg/mL (MIC ≤1 μg/mL) can achieve the target area under the concentration-time curve (AUC)/MIC ratio of >400, which is a suitable target to attain successful clinical efficacy in adults (3–6). Although the latest IDSA guidelines note that the AUC-guided administration strategy based on the Bayesian method may be the best method for individualized vancomycin therapy (7), it is still necessary and feasible to use the trough concentrations for medication guidance owing to the relative difficulty in calculating and evaluating the AUC_24_ for individual patients in many hospitals in developing countries.

In pediatric patients treated with vancomycin, the clearance rate is higher and the vancomycin half-life time is shorter than those in adults (8), and a previous study reported that a vancomycin trough concentration of 10 μg/mL achieved an AUC/MIC >400 in more than 90% of children infected by Gram-positive pathogens, with a vancomycin MIC of ≤1 μg/mL (9). Therefore, referring to the trough concentration range for adults during the treatment of children is controversial and may not be appropriate. However, few studies have evaluated the relationship between clinical efficacy and vancomycin trough concentrations in children, with even less data on the AUC. Therefore, the optimal vancomycin trough level for children is still unclear (10, 11), causing substantial clinical challenges in the safe and effective use of vancomycin in children (12–14).

We conducted this study to evaluate the clinical efficacy and safety of different vancomycin trough concentrations in children via meta-analysis to determine the correlation between vancomycin serum trough concentrations and clinical outcomes, provide evidence for the rational administration of vancomycin in children and improve the level of rational drug use.

## RESULTS

### Literature search and study selection.

**FIG 1:**
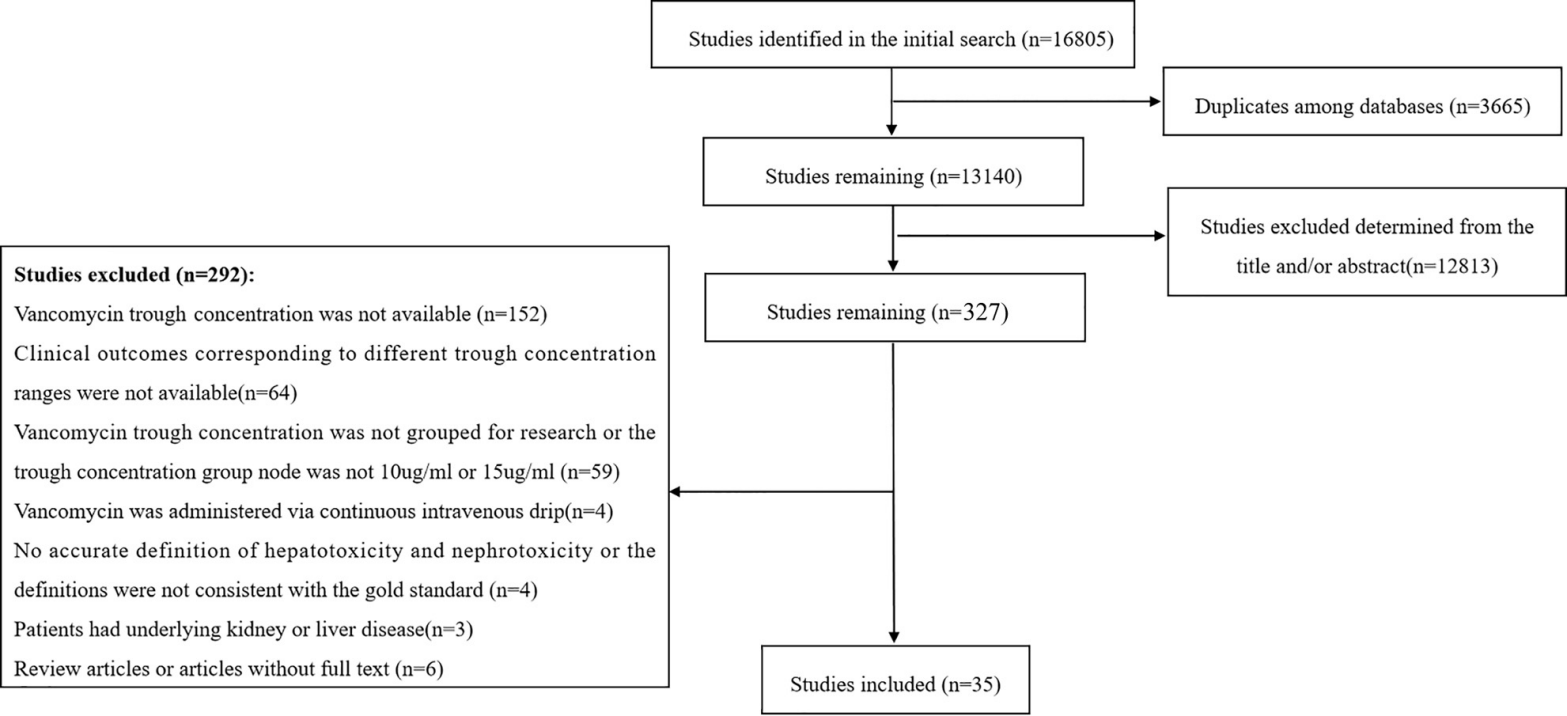
Flow diagram of the study selection process.

The initial search yielded 16805 potentially relevant articles, including 9629 records in English and 7176 records in Chinese. Finally, 35 studies (12–46) were enrolled in the systematic review and meta-analysis. The entire screening and selection process is shown in Fig. 1.

### Basic characteristics of the included studies.

A total of 35 studies (12–46) involving 4820 patients were included; 3059 patients had a vancomycin serum trough concentration <10 μg/mL and 1761 patients had trough concentration ≥10 μg/mL. When designing the experimental group and the control group in each study, the basic conditions of the patients and the types of infections were considered, and the baselines of the two groups were comparable. The types of infections included pneumonia, meningitis, peritonitis, sepsis, skin and soft tissue infection, bacteremia, urinary tract infection, abdominal infection, and central nervous system infection. The standard vancomycin dose recommended by the clinical guidelines was administered intravenously in children. The basic characteristics of the included studies were presented in Table 1.

**TABLE 1:** Characteristics and quality assessments of the included studies

Study	Year of study	Country	Age	Study design	Infection sites	Participants		Dosages	Durations	Outcomes^*a*^	NOS/adjusted Jadad scale
						<10 μg/mL	≥10 μg/mL				
Yoo 2021 (45)	2010–2018	Korea	2 mo −18 yrs	Single-center retrospective study	MRSA bacteremia, severe pneumonia, etc.	52	21	Standard dosage^*b*^	≥48 h	(i), (v), (vi)	6
Shin 2020 (41)	2008–2016	Korea	≤12 yrs	Single-center retrospective study	SSSI^*c*^, UTI, severe pneumonia, etc.	120	30	Standard dosage	≥72 h	(i), (ii), (v)	7
Tu 2020 (15)	2019	China	5 h–13.5 yrs	Single-center prospective study	Severe pneumonia, NEC, SSSI, etc.	57	40	10-15 mg/kg q6-12 h	1 w–24 d	(ii), (iii), (iv)	5
Wang 2020 (28)	2018–2020	China	0–15 yrs	Single-center retrospective study	Sepsis, cellulitis, suppurative meningitis, etc.	9	26	15-60 mg/kg/d	≥4 doses	(i)	7
Sridharan 2019 (44)	2016–2019	Saudi Arabia	<18 yrs	Single-center retrospective study	Severe pneumonia, CNS infection, etc.	96	156	Standard dosage	5.5 d	(ii), (v)	5
Zhu 2019 (16)	2018	China	≤12 yrs	Single-center retrospective study	Severe pneumonia caused by MRSA or G+ bacteria, osteomyelitis, meningitis, UTI, etc.	105	53	1-3 g/d q8-12 h	>5 d	(i), (vi)	7
Yin 2019 (31)	2014–2018	China	<3 mo	Single-center retrospective study	Sepsis caused by G+ bacteria	37	18	15 mg/kg q8 h-qd	≥72 h	(i)	7
Tao 2019 (26)	2014–2015	China	0–28 d	Single-center prospective study	NA	48	63	10-15 mg/kg q8-12 h	≥3 d	(ii)	6
Xu 2018 (29)	2015–2017	China	≤28 d	Single-center prospective study	Septicemia caused by MRSA	8	42	10-15 mg/kg q8-12 h	7–14 d	(i), (ii), (iv), (vi)	7
Shen 2018 (43)	2013–2017	China	≤18 yrs	Single-center retrospective study	CNS infections, febrile neutropenia, abdominal infection, etc.	97	73	Standard dosage	≥48 h	(ii)	7
Qing 2018 (21)	2015–2017	China	7 h–29 d	Single-center prospective study	Severe pneumonia, sepsis, meningitis, etc.	11	35	Standard dosage	≥4 doses	(ii), (iii), (iv)	6
Hsu 2017 (34)	2007–2014	USA	<18 yrs	Multicenter retrospective study	Osteoarthritis caused by MRSA, SSSI, severe pneumonia or CNS infections	105	38	Standard dosage	≥72 h	(vi)	7
Bhargava 2017 (13)	2008–2012	USA	Premature infants	Single-center retrospective study	Sepsis, NEC, etc.	72	38	Standard dosage	≥5 d	(ii)	9
Yoo 2017 (46)	2010–2014	Korea	<18 yrs	Single-center retrospective study	Bacteremia caused by MRSA	35	10	Standard dosage	≥48 h	(i), (v), (vi)	6
Lv 2017 (19)	2014–2016	China	6 h–30 d	Single-center prospective study	Sepsis, severe pneumonia, peritonitis, microbial meningitis, etc.	136	24	15 mg/kg q8-12 h	7–30 d	(ii), (iii), (iv)	7
Wang 2017 (27)	2015	China	0–30 d	Single-center prospective study	Severe pneumonia, sepsis, etc.	10	33	Standard dosage	≥4 doses	(ii), (iii), (iv)	6
Wei 2016 (12)	2007–2015	China	<18 yrs	Single-center retrospective study	Respiratory infection, BSI, CNS infection, SSSI, etc.	141	24	Standard dosage	8–18 d	(i), (ii)	9
Bonazza 2016 (33)	2011–2014	Canada	<18 yrs	Single-center prospective RCT	NA	153	112	Standard dosage	≥3 doses	(ii)	5
McNeil 2016 (38)	2003–2013	USA	1.2 mo–115.7 mo	Single-center retrospective study	Bacteremia, BSI, infective endocarditis	55	72	Standard dosage	≥96 h	(ii), (v)	9
McNeil 2017 (37)	2011–2014	USA	<18 yrs	Single-center retrospective study	AHO and septic osteoarthritis	20	24	Standard dosage	≥96 h	(ii)	9
Yan 2016 (30)	2013–2015	China	0–18 yrs	Single-center retrospective study	NA	40	16	Standard dosage	≥3 doses	(i)	6
Ren 2016 (22)	2010–2015	China	<18 yrs	Single-center retrospective study	Meningitis, severe pneumonia, sepsis, etc.	41	60	Standard dosage	≥4 doses	(i)	6
Guo 2016 (14)	2012–2015	China	7 h–28 d	Single-center retrospective study	Severe pneumonia, meningitis, sepsis, etc.	143	17	10-15 mg/kg q8-12 h	7–30 d	(ii), (iii), (iv)	9
Tang 2016 (23)	2009–2015	China	0–28 d	Single-center prospective RCT	G+ sepsis	42	66	10-15 mg/kg q8-12 h	10–14 d	(i), (vi)	5
Tang 2016 (24)	2012–2013	China	0–28 d	Single-center retrospective study	G+ sepsis	19	54	10-15 mg/kg q8-12 h	7–14 d	(i), (vi)	9
Ringenberg 2015 (40)	2010–2012	USA	Neonates	Multicenter retrospective study	Severe pneumonia, UTI, sepsis, SSSI	123	48	Standard dosage	≥3 doses	(ii)	6
Li 2015 (17)	2011–2014	China	0–28 d	Single-center retrospective study	Respiratory infection, BSI, etc.	38	41	10-15 mg/kg q8-12 h	4–41 d	(i)	9
Tang 2015 (25)	2012–2014	China	0–28 d	Single-center retrospective study	Severe pneumonia, microbial meningitis, sepsis, SSSI, etc.	146	28	10-15 mg/kg q8-12 h	7–30 d	(ii), (iii), (iv)	9
Sinkeler 2014 (42)	2009–2012	Netherlands	Premature infants	Single-center retrospective study	Sepsis, CNS infections, NEC	53	59	Standard dosage	≥4 doses	(v)	6
Jennifer 2014 (35)	2003–2011	USA	3 mo–21 yrs	Multicenter retrospective study	NA	400	280	Standard dosage	≥48 h	(ii)	7
Liu 2014 (18)	2011–2014	China	2 d–9 yrs	Single-center retrospective study	NA	152	16	10-15 mg/kg q8-12 h	7–28 d	(iii), (iv)	9
Ahmed 2013 (39)	2010–2012	Egypt	1 wk–15 yrs	Single-center retrospective study	Bacteremia, severe pneumonia, meningitis, SSSI, arthritis, endocarditis	166	99	Standard dosage	≥48 h	(ii)	7
Zhang 2013 (32)	2008–2012	China	7 h–9 yrs	Single-center retrospective study	Severe pneumonia, septicemia, meningitis, peritonitis, SSSI, septic arthritis, etc.	213	22	15 mg/kg q8-12 h	7–30 d	(ii), (iii), (iv)	9
Peng 2013 (20)	2011–2012	China	0–13 yrs	Single-center retrospective study	Severe pneumonia, sepsis, septicemia and bronchopneumonia, cellulitis, etc.	109	21	Standard dosage	≥72 h	(i)	9
Machado 2001 (36)	1995–1997	Brazil	38–44 wks	Single-center retrospective study	Sepsis	7	2	Standard dosage	14d	(i)	6

a (i) clinical efficacy, (ii) nephrotoxicity, (iii) hepatotoxicity, (iv) ototoxicity, (v) mortality, (vi) microbial clearance.

b Standard dosage: the guideline recommendation of 40 mg/kg/d; for severe infection, vancomycin can be administered at a dosage of 60 mg/kg/d (3, 7).

c SSSI: skin and skin structure infection, UTI: urinary tract infection, NEC: necrotizing enterocolitis, CNS: central nervous system, NA: not available, BSI: bloodstream infection, AHO: acute hematogenous osteomyelitis.

### Quality of the studies.

Of the 35 included studies, 2 studies (23, 33) were randomized controlled trials (RCTs), and the adjusted Jadad scale showed that they were high-quality studies (score of 5). The remaining 33 studies were retrospective and assessed via the Newcastle-Ottowa scale (NOS). Ultimately, 11 studies (12–14, 17, 18, 20, 24, 25, 32, 37, 38) had NOS scores of 9, and the other 22 studies (15, 16, 19, 21, 22, 26–31, 34–36, 39–46) had scores ranging from 5 to 7. Therefore, the quality of all the studies was acceptable, as shown in Table 1.

### Results of the meta-analysis.

**FIG 2:**
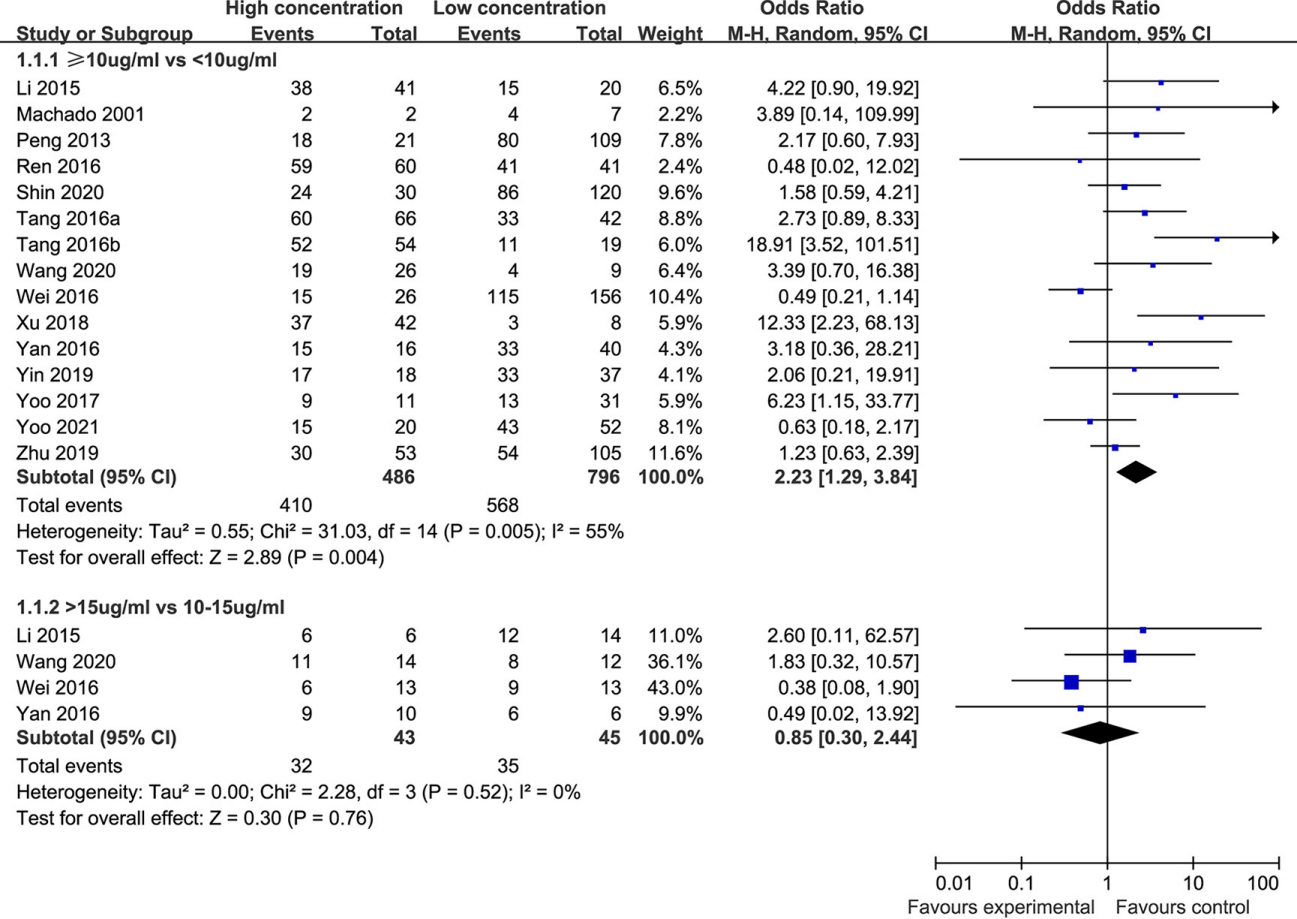
Clinical efficacy levels in different trough concentration groups.

**(i) Clinical efficacy.** Fifteen studies (12, 16, 17, 20, 22–24, 28–31, 37, 41, 45, 46) compared the clinical efficacy between the two vancomycin trough concentration groups (<10 μg/mL and ≥10 μg/mL). The included studies exhibited significant heterogeneity (*P* = 0.005, I^2^ = 55%). Therefore, the random-effect model was used for analysis. As shown in Fig. 2, clinical efficacy was significantly lower in the trough concentration <10 μg/mL group (71.4%) than in the trough concentration ≥10 μg/mL group (84.4%), and the difference between the two groups was statistically significant [OR: 2.23, 95% CI: 1.29 to 3.84, *P* = 0.004].

Four studies (12, 17, 28, 30) compared the clinical efficacy between groups with a trough concentration of 10 to 15 μg/mL and a trough concentration of >15 μg/mL. There was no significant heterogeneity among the included studies (*P* = 0.52, I^2^ = 0%). Although the clinical efficacy in the 10 to 15 μg/mL group (77.8%) was slightly elevated compared with that in the >15 μg/mL group (74.4%), there was no statistically significant difference between the two groups [OR: 0.85, 95% CI: 0.30 to 2.44, *P* = 0.76], as shown in Fig. 2.

**FIG 3:**
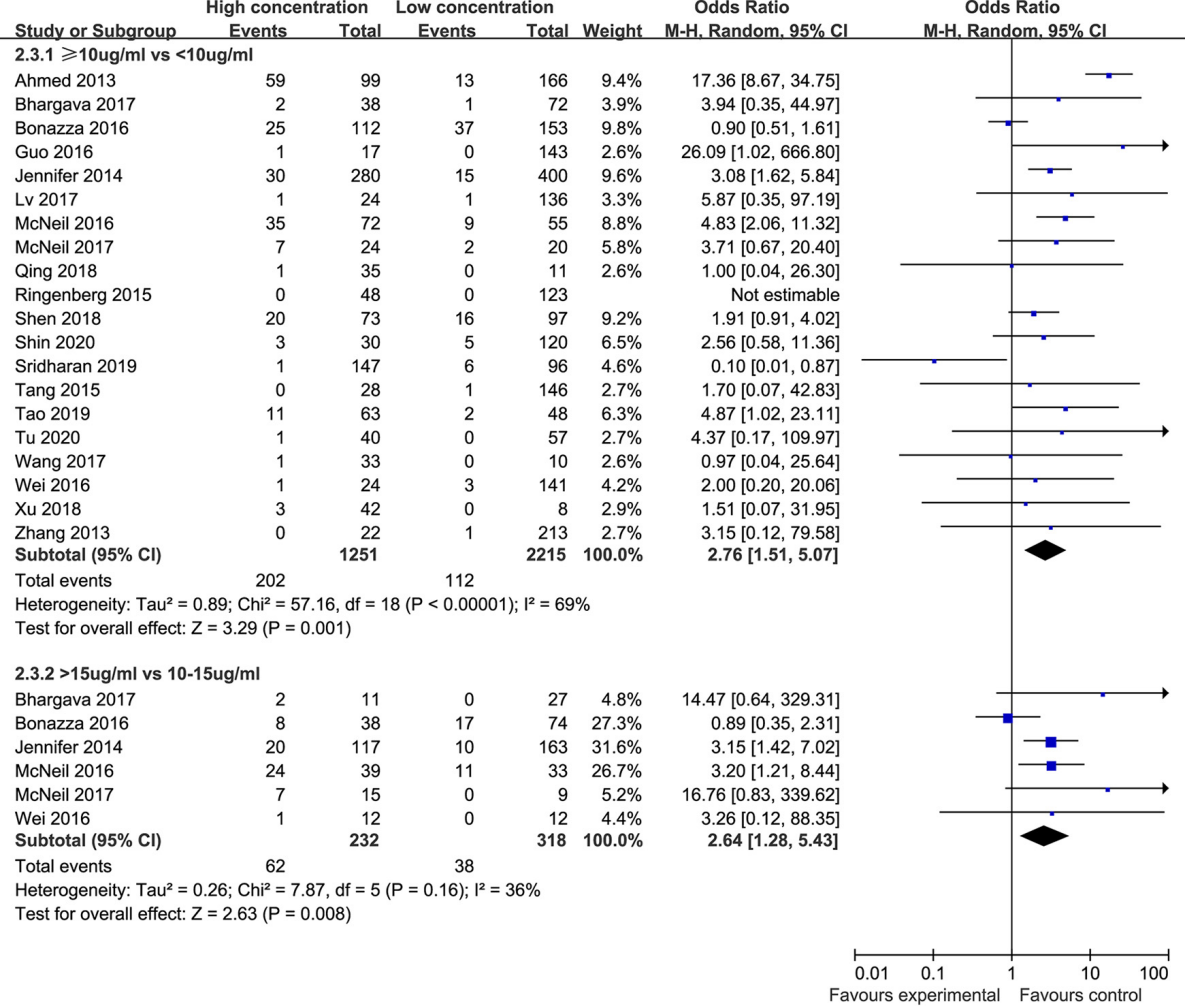
Nephrotoxicity rates in different trough concentration groups.

**(ii) Nephrotoxicity.** Twenty studies (12–15, 19, 21, 25–27, 29, 32, 33, 35, 37–41, 43, 44) compared the incidence of nephrotoxicity in children with vancomycin trough concentrations <10 μg/mL and ≥10 μg/mL. The included studies displayed significant heterogeneity (*P* < 0.00001, I^2^ = 69%), so the random-effect model was used for analysis. The meta-analysis showed that the incidence of nephrotoxicity in the trough concentration <10 μg/mL group (5.1%) was significantly lower than that in the trough concentration ≥10 μg/mL group (16.1%), and the difference between the two groups was statistically significant [OR: 2.76, 95% CI: 1.51 to 5.07, *P* = 0.001], as shown in Fig. 3.

Six studies (12, 13, 33, 35, 37, 38) compared the incidence of nephrotoxicity in children with vancomycin trough concentrations of 10 to 15 μg/mL and >15 μg/mL. As shown in Fig. 3, there was no significant heterogeneity among the included studies (*P* = 0.16, I^2^ = 36%). The results showed that the incidence of nephrotoxicity in the 10 to 15 μg/mL group (11.9%) was significantly lower than that in the >15 μg/mL group (26.7%), and the difference between the two groups was statistically significant [OR: 2.64, 95% CI: 1.28 to 5.43, *P* = 0.008].

**FIG 4:**
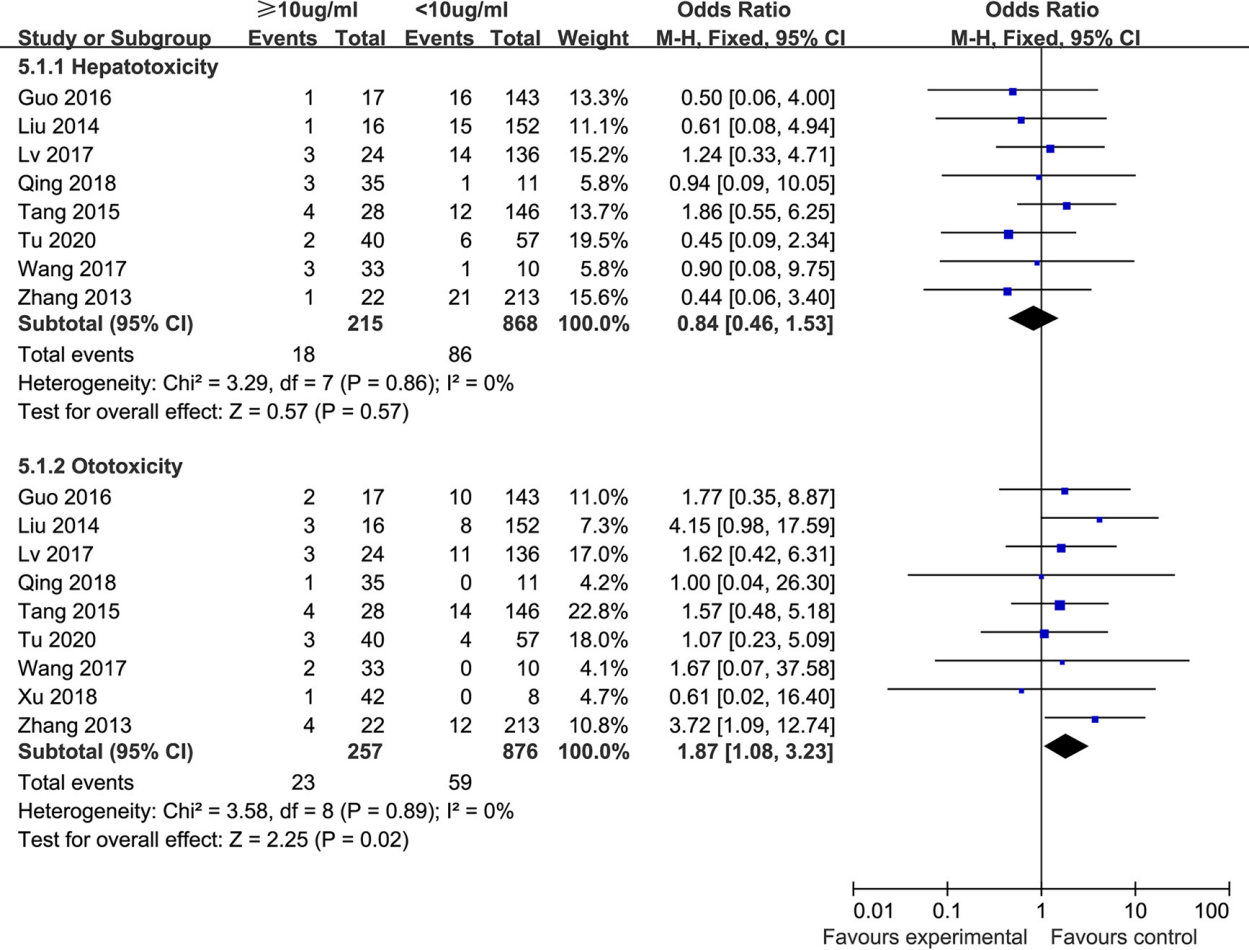
Hepatotoxicity and ototoxicity rates in different trough concentration groups.

**(iii) Hepatotoxicity and ototoxicity.** Eight studies (14, 15, 18, 19, 21, 25, 27, 32) compared the incidence of hepatotoxicity in children with vancomycin trough concentrations <10 μg/mL and ≥10 μg/mL. The included studies exhibited no significant heterogeneity (*P* = 0.86, I^2^ = 0%), so the fixed-effect model was used for analysis. As shown in Fig. 4, the meta-analysis showed that the incidence of hepatotoxicity in the trough concentration <10 μg/mL group (9.9%) was slightly higher than that in the trough concentration ≥10 μg/mL group (8.4%), but there was no statistically significant difference between the two groups [OR: 0.84, 95% CI: 0.46 to 1.53, *P* = 0.57].

Nine studies (14, 15, 18, 19, 21, 25, 27, 29, 32) compared the incidence of ototoxicity in children with vancomycin trough concentrations <10 μg/mL and ≥10 μg/mL. There was no significant heterogeneity among the included studies (*P* = 0.89, I^2^ = 0%); hence, the fixed-effect model was used for analysis. The results showed that the incidence of ototoxicity in the trough concentration <10 μg/mL group (6.7%) was significantly lower than that in the trough concentration ≥10 μg/mL group (8.9%), and the difference between the two groups was statistically significant [OR: 1.87, 95% CI: 1.08 to 3.23, *P* = 0.02], as shown in Fig. 4.

**FIG 5:**
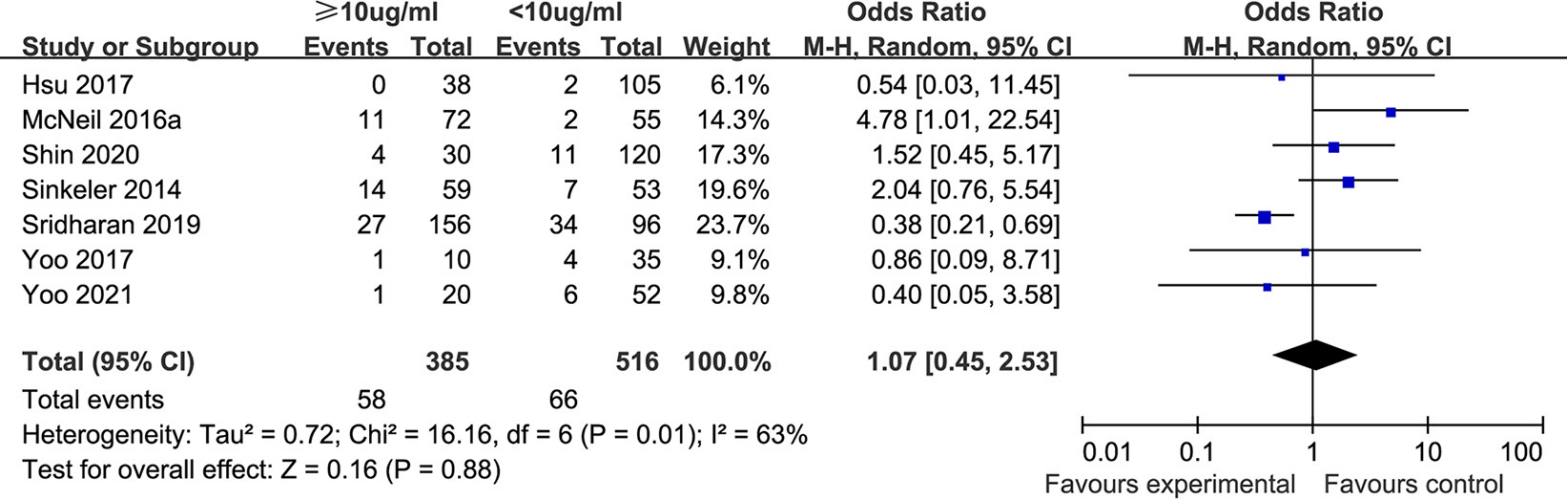
All-cause mortality rates in different trough concentration groups.

**(iv) All-cause mortality.** Seven studies (34, 38, 41, 42, 44–46) compared all-cause mortality between the vancomycin trough concentration <10 μg/mL group and the trough concentration ≥10 μg/mL group. There was significant heterogeneity among the included studies (*P* = 0.01, I^2^ = 63%), so the random-effect model was used for analysis. The meta-analysis results showed that the all-cause mortality rate in the trough concentration <10 μg/mL group (12.8%) was slightly lower than that in the trough concentration ≥10 μg/mL group (15.1%), but there was no significant difference between the two groups [OR: 1.07, 95% CI: 0.45 to 2.53, *P* = 0.88], as shown in Fig. 5.

**FIG 6:**
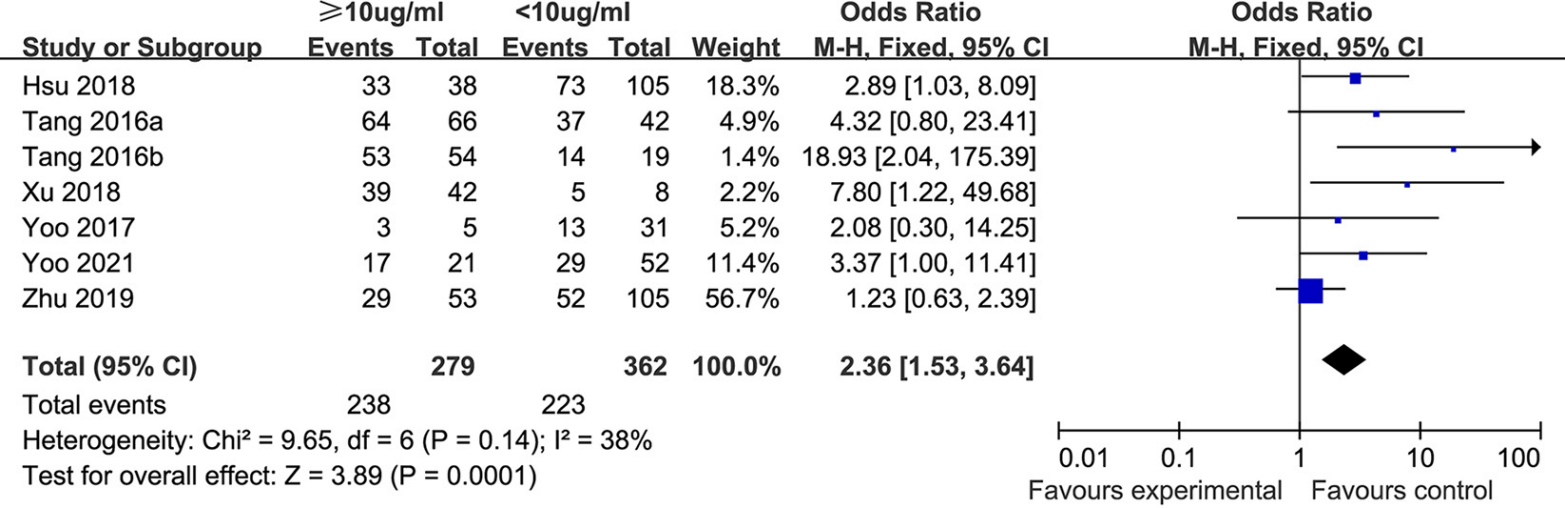
Microbial clearance rates in different trough concentration groups.

**(v) Microbial clearance.** Seven studies (16, 23, 24, 29, 34, 45, 46) compared the microbial clearance rate between the vancomycin trough concentration <10 μg/mL group and the vancomycin trough concentration ≥10 μg/mL group. There was no significant heterogeneity among the included studies (*P* = 0.14, I^2^ = 38%); hence, the fixed-effect model was used for analysis. As shown in Fig. 6, the forest plot showed that the microbial clearance rate in the trough concentration <10 μg/mL group (61.6%) was lower than that in the trough concentration ≥10 μg/mL group (85.3%), and the difference between the two groups was statistically significant [OR: 2.36, 95% CI: 1.53 to 3.64, *P* = 0.0001].

### Publication bias.

Egger's test was performed for the two outcomes, namely, the clinical efficacy rate and the incidence of nephrotoxicity, to determine whether their publication bias existed in the included literature. The results showed that there was no publication bias in the included literature for clinical efficacy (<10 μg/mL versus ≥10 μg/mL, *P* = 0.108) and nephrotoxicity (<10 μg/mL versus ≥10 μg/mL, *P* = 0.901). The remaining outcomes were not subjected to Egger's test because the accumulated number of eligible studies was <10.

### Sensitivity analysis.

Sensitivity analysis was carried out by examining the influence of a single study on the pooled effect size. If the results before and after the sensitivity analysis showed no statistical significance, the results of the meta-analysis were considered robust. Otherwise, the results indicated that there were potentially important factors related to the intervention measures that affected the reliability of the results. Sensitivity analysis was performed to evaluate the clinical efficacy (Fig. S1A), nephrotoxicity (Fig. S1B), mortality (Fig. S1C) and microbial clearance (Fig. S1D) in children with vancomycin trough concentrations <10 μg/mL and ≥10 μg/mL, and the results showed that the pooled effect size for these four outcomes did not change substantially compared with the previous results, suggesting that the meta-analysis results were credible.

## DISCUSSION

Vancomycin is widely used for the treatment of infectious diseases caused by Gram-positive pathogens in children, such as suppurative meningitis, bacteremia, osteomyelitis, and necrotizing enterocolitis. The previous IDSA guidelines published in 2011 recommend that vancomycin trough concentrations are closely related to clinical outcomes in adults and can predict adverse reactions as well as the clinical efficacy of vancomycin (3). Several studies have shown that vancomycin trough concentration monitoring can improve clinical efficacy and reduce nephrotoxicity in children infected with Gram-positive pathogens, similar to adults (6, 45–47). Therefore, it is necessary to monitor the serum trough concentrations in children. However, currently, there is no recommended vancomycin trough concentration range for children, so medical personnel must refer only to the adult standard, and according to published reports, the trough concentrations in most children are low and do not reach the adult standard after the administration of standard doses of vancomycin (3, 14, 48).

The clinical efficacy and safety of vancomycin at different trough concentrations in children were compared in our meta-analysis. The results indicated that the incidences of nephrotoxicity and ototoxicity in children in the <10 μg/mL group were significantly lower than those in children in the ≥10 μg/mL group, and the incidence of nephrotoxicity in the 10 to 15 μg/mL group was lower than that in the >15 μg/mL group, consistent with the published literature (12–14, 25, 35, 37). Therefore, the higher the trough concentration of vancomycin is, the greater the risk of nephrotoxicity and ototoxicity. That is, the incidences of nephrotoxicity and ototoxicity were the highest in the >15 μg/mL group, followed by the 10 to 15 μg/mL group and the lowest incidence was observed in the <10 μg/mL group. Furthermore, we revealed that clinical efficacy rates and microbial clearance in the ≥10 μg/mL group were significantly higher than those in the <10 μg/mL group, but the clinical efficacy in the 10 to 15 μg/mL group showed no significant difference compared with that in the >15 μg/mL group. Therefore, maintaining a vancomycin trough concentration at 10 to 15 μg/mL could achieve effective clinical efficacy in children. Interestingly, the all-cause mortality in the ≥10 μg/mL group was comparable to that in the <10 μg/mL group, which may be because the lower clinical efficacy in the <10 μg/mL group lead to poor infection control and even death, even if the low incidence of adverse reactions; however, higher trough concentrations, especially trough concentrations >20 μg/mL, can lead to serious and fatal adverse reactions, even if the better clinical efficacy (35). Moreover, the vancomycin serum trough concentrations had a low correlation with hepatotoxicity, which may be because vancomycin is not metabolized in the body, and 90% of the given dose is eliminated by the kidney in the prototype form so it has little effect on the liver (49). Regarding the clinical use of drugs, both safety and efficacy should be considered. Although a trough concentration <10 μg/mL was associated with lower rates of nephrotoxicity, ototoxicity, and hepatotoxicity in this meta-analysis, it was also associated with lower clinical efficacy than trough concentrations ≥10 μg/mL. A trough concentration >15 μg/mL has worse safety, but clinical efficacy is the same as that of trough concentrations of 10 to 15 μg/mL. Maintaining the trough concentration at 10 to 15 μg/mL can ensure the best clinical efficacy, and the incidence of adverse drug reactions is relatively low and within a controllable range. Consequently, our findings provide strong evidence supporting that maintaining the vancomycin trough concentrations in children at 10 to 15 μg/mL can improve the clinical efficacy, and avoidance of trough concentrations >15 μg/mL can reduce the incidence of adverse effects in children.

The latest IDSA guidelines recommend using AUC/MIC to guide the application of vancomycin. Based on research data in adults, it is recommended that AUC/MIC should be maintained at 400 to 600 (MIC = 1 μg/mL) in the treatment of severe MRSA infection to achieve clinical efficacy and ensure patient safety. It is suggested that vancomycin treatment drug monitoring under the guidance of AUC should be carried out for children of all ages (7). However, first, the Bayesian method for the calculation of AUC requires an accurate understanding of population modeling, while the PK data of vancomycin in Chinese children have rarely been reported. In addition, AUC calculation requires Bayesian software tools and personnel training to implement in clinical practice. Therefore, the Bayesian method is difficult to perform in many hospitals in developing countries, including our hospital, which is a large tertiary teaching hospital, because of a lack of tools and required knowledge. The first-order PK equations to calculate vancomycin AUC would be the next preferred method, but of which there are certain restrictions in children on account of needing collecting 2 steady-state samples to calculate AUC (50, 51). Second, according to a systematic review of the correlation between vancomycin trough concentrations and AUC/MIC in children, there is still a lack of research data on the target vancomycin trough concentrations in children, not to mention the target AUC/MIC. Furthermore, few studies have explored the relationships among vancomycin trough concentrations, AUC/MIC, and clinical outcomes. Hence, more research is needed to determine whether the AUC/MIC target values for adults apply to children (52). Importantly, the updated vancomycin guidelines published by the Chinese Pharmacological Society in Clinical Infectious Diseases in 2020 note that the trough concentrations and AUC_24_ are recommended at the same strength (51). Therefore, monitoring trough concentrations may not be the most accurate, but it is indeed the most suitable and accessible method in developing countries.

Currently, a few pharmacokinetic and pharmacodynamic (PK/PD) studies have shown a correlation between AUC/MIC and vancomycin trough concentrations (9, 35, 48). A study conducted by Kishk et al. (48) demonstrated that an AUC/MIC of 400 in children was related to a trough concentration of 11 μg/mL using a trapezoidal method to calculate the AUC. A systematic review (52) revealed that trough concentrations of 6 to 10 mg/L were appropriate for achieving an AUC_24_ ≥ 400 in most general hospitalized pediatric patients, and a prospective observational study in critically ill children showed that a trough concentration of 7 mg/L corresponded to an AUC_24_ of 400 (53). Moreover, the results of a retrospective cohort study (35) included in our research demonstrated that trough concentrations ≥15 μg/mL and an AUC ≥800 were independently associated with a significantly increased risk of vancomycin-associated nephrotoxicity. Based on these findings, the target range of 10 to 15 μg/mL obtained in our study can result in the AUC/MIC reaching the expected range, improving clinical efficacy and reducing adverse reactions. Therefore, although the optimal AUC/MIC target range was not analyzed, our results identified the optimal target vancomycin trough concentration range in pediatric patients based on clinical efficacy and adverse drug reactions, which is indicative of the AUC/MIC.

This study has several strengths. First, based on a comprehensive literature search, we enrolled more studies than other meta-analyses and included studies focused on the clinical efficacy and safety of vancomycin in Chinese children for the first time. Second, to our knowledge, our meta-analysis was the first to directly compare vancomycin trough concentrations between 10 and 15 μg/mL and other trough ranges, rather than by indirect comparison, which concludes that vancomycin trough concentrations of 10 to 15 μg/mL are the most suitable concentration range for children more robust and reliable. Last, this is the first meta-analysis to reveal the associations between vancomycin through concentrations and hepatotoxicity, ototoxicity, and microbial clearance.

Our research has several limitations. Only 2 RCTs were included, and the rest were retrospective studies. Most of the included studies were single-center explorations, and their results may contain bias. However, the current results were not significantly altered during sensitivity analysis, indicating that the included studies lacked heterogeneity and that our findings were relatively robust. The included studies lacked long-term follow-up data. Therefore, it was difficult to analyze long-term mortality. The efficacy definition varied among the enrolled studies. There were PK differences among neonates and children outside the neonatal period, but due to the limitations of the included studies, we could neither conduct a subgroup analysis to evaluate the correlation between clinical efficacy and safety and the trough concentrations in neonates nor remove neonatal patients from this analysis. Therefore, the conclusions of our study should be cautiously applied to neonates, and the recommended optimum vancomycin trough concentrations for neonates remain to be determined by further research. In addition, some other subgroup analyses for various types of confounders, such as infectious types and bacteria, could not be performed due to a lack of studies.

In conclusion, our meta-analysis highlights the findings that vancomycin trough concentrations are significantly related to clinical efficacy and safety in children. In terms of adverse reactions, the incidences of nephrotoxicity and ototoxicity were the highest in the >15 μg/mL group, followed by the 10 to 15 μg/mL group, and the lowest incidence was observed in the <10 μg/mL group. As far as clinical efficacy, a trough concentration <10 μg/mL is the worst, and a trough concentration >15 μg/mL is almost equivalent to that of a trough concentration of 10 to 15 μg/mL. Hence, maintaining the vancomycin trough concentrations at 10 to 15 μg/mL may improve the clinical efficacy in children. Additionally, avoidance of trough concentrations >15 μg/mL can reduce the incidence of adverse effects. We look forward to prospective, large-scale randomized controlled studies in children to evaluate the relationship between vancomycin trough concentrations and clinical outcomes and provide evidence for optimal surveillance strategies to optimize vancomycin use in children.

## MATERIALS AND METHODS

### Inclusion criteria.

Inclusion criteria included the following. The population: patients aged ≤18 years who received intravenous vancomycin therapy and had measured steady-state trough concentrations. Intervention and comparison: vancomycin trough concentrations of 10 ug/ml or 15 ug/ml were used as the nodes; concentrations were divided into two trough concentration ranges of <10 ug/ml and ≥10 ug/ml if only the 10 ug/ml node was present. Concentrations were divided into three trough concentration ranges of <10 ug/ml, 10 to 15 ug/ml, and >15 ug/ml if both the 10 ug/ml and 15 ug/ml nodes were present simultaneously. Clinical outcomes corresponding to the two or three different trough concentration ranges defined herein were extracted in the literature. The type of infection, dose, and duration of treatment were not limited.

Outcomes: clinical efficacy, nephrotoxicity, hepatotoxicity, ototoxicity, all-cause mortality, and microbial clearance. Clinical efficacy was defined as at least 3 of 4 clinical items (clinical symptoms, signs, laboratory parameters, and pathogens) returning to within their normal ranges or bacteremia clearance without recurrence. Nephrotoxicity was defined as an increase in the serum creatinine level of at least 0.5 mg/dl or a 50% increase from the baseline level (7). Hepatotoxicity was defined as the concurrent elevation of aspartate aminotransferase (AST), alkaline phosphatase (ALP), and total bilirubin (TB) levels, with at least one of them exceeding two times the upper limit of the corresponding baseline, or as a 2-fold increase in the serum alanine aminotransferase (ALT) or conjugated bilirubin (CB) level from baseline. Viral hepatitis and other causes of hepatotoxicity were excluded by serum hepatitis antigen-antibody system examination and the patient’s medical history (15). Microbial clearance was defined as clearance and presumed clearance while receiving therapy or within 30 days after treatment with vancomycin (nonclearance was defined as the positive culture of infectious pathogens from the original infection site after treatment; for clinically invalid cases, if an additional sample could not be obtained and bacterial reculture was not performed, and it is defined as assuming no clearance. Clearance was defined as the absence of infectious pathogens from the original infected site after treatment. Presumed clearance was defined as a clinically effective curative effect, but an additional sample was not obtained, and the bacterial culture was not rechecked after treatment) (16). Study design: published randomized controlled trials (RCTs) and prospective or retrospective studies.

### Exclusion criteria.

Exclusion criteria included (i) an unavailable vancomycin trough concentration; (ii) vancomycin trough concentrations being not stratified in the research, or trough concentration group nodes not 10 μg/mL nor 15 μg/mL; (iii) lack of assessment of trough concentration ranges defined in our paper against the corresponding outcomes; (iv) vancomycin was administered via a continuous intravenous drip; (v) lack of accurate definitions of hepatotoxicity and nephrotoxicity, definitions not consistent with the gold standard, or enrolled patients with underlying kidney or liver disease; (vi) only pharmacokinetic analyses; (vii) duplicated publications, case reports and a publication in a language other than Chinese or English.

### Search strategy.

All studies were identified by a systematic review of databases, including PubMed, Elsevier, Web of Science, EMBASE, Medline, clinicaltrials.gov, the Cochrane Library, and three Chinese databases (Wanfang Data, China National Knowledge Infrastructure, and SINOMED) up to December 2021 using the following terms: vancomycin, norvancomycin, vancocin, and concentration. A search method combining subject headings and free text was used, and adjustments were made according to the specific databases. The reference lists of the included papers and previous reviews were manually screened to identify additional studies.

### Data extraction.

Two investigators (LC and SYY) independently screened the publications, extracted the data, and cross-checked the results. Any disagreements were resolved by consensus or consultation with a third investigator. The following data were extracted independently: (i) basic information of the publication (first author, publication year, and type of study); (ii) clinical characteristics of the patients (age, number of patients, and types of infections); (iii) intervention and control measures; (iv) clinical outcomes (clinical efficacy, nephrotoxicity, hepatotoxicity, ototoxicity, mortality, and microbial clearance); and (v) quality assessment indicators.

### Quality assessment.

RCTs were assessed using the adjusted Jadad scale. Assessment parameters included the randomization method, allocation concealment, blinding, and patient loss to follow-up or withdrawal. The total score was 7; studies with a score between 1 and 3 were considered low-quality studies, while those with a score between 4 and 7 were considered high-quality studies. Retrospective studies were evaluated with the Newcastle-Ottawa scale (NOS) (54), which assesses the representativeness of participants, comparability of participants, follow-up and assessment of follow-up sufficiency, and patient loss to follow-up or withdrawal. Studies with a score between 5 and 9 were considered to have less bias and were included in the meta-analysis.

### Statistical analysis.

Meta-analysis was performed using RevMan software (version 5.3). The dichotomous outcomes are expressed as the odds ratio (OR) and 95% confidence interval (CI). Heterogeneity was measured using the Cochrane Q test. When *P* > 0.1 and I^2^ ≤ 50%, there was no significant difference in the heterogeneity among studies, and a fixed-effect model was used for combined analysis. Otherwise, a random-effect model was used for combined analysis. Stata 15.0 software was used to perform a sensitivity analysis of the outcomes with high heterogeneity in the included literature. Egger’s test was used for publication bias assessment of clinical outcomes that were included in a sufficient number of studies (at least 10 studies). *P* < 0.05 were considered statistically significant.
