# Supplementary material for: Relationship between Vancomycin Trough Serum Concentrations and Clinical Outcomes in Children: a Systematic Review and Meta-Analysis
Source: Antimicrob Agents Chemother. 2022 Jul 13;66(8):e00138-22. doi: 10.1128/aac.00138-22 (PMC9380573; doi:10.1128/aac.00138-22)
Supplement: Supplemental file 1 — Fig. S1. Download aac.00138-22-s0001.pdf, PDF file, 0.2 MB [file aac.00138-22-s0001.pdf]

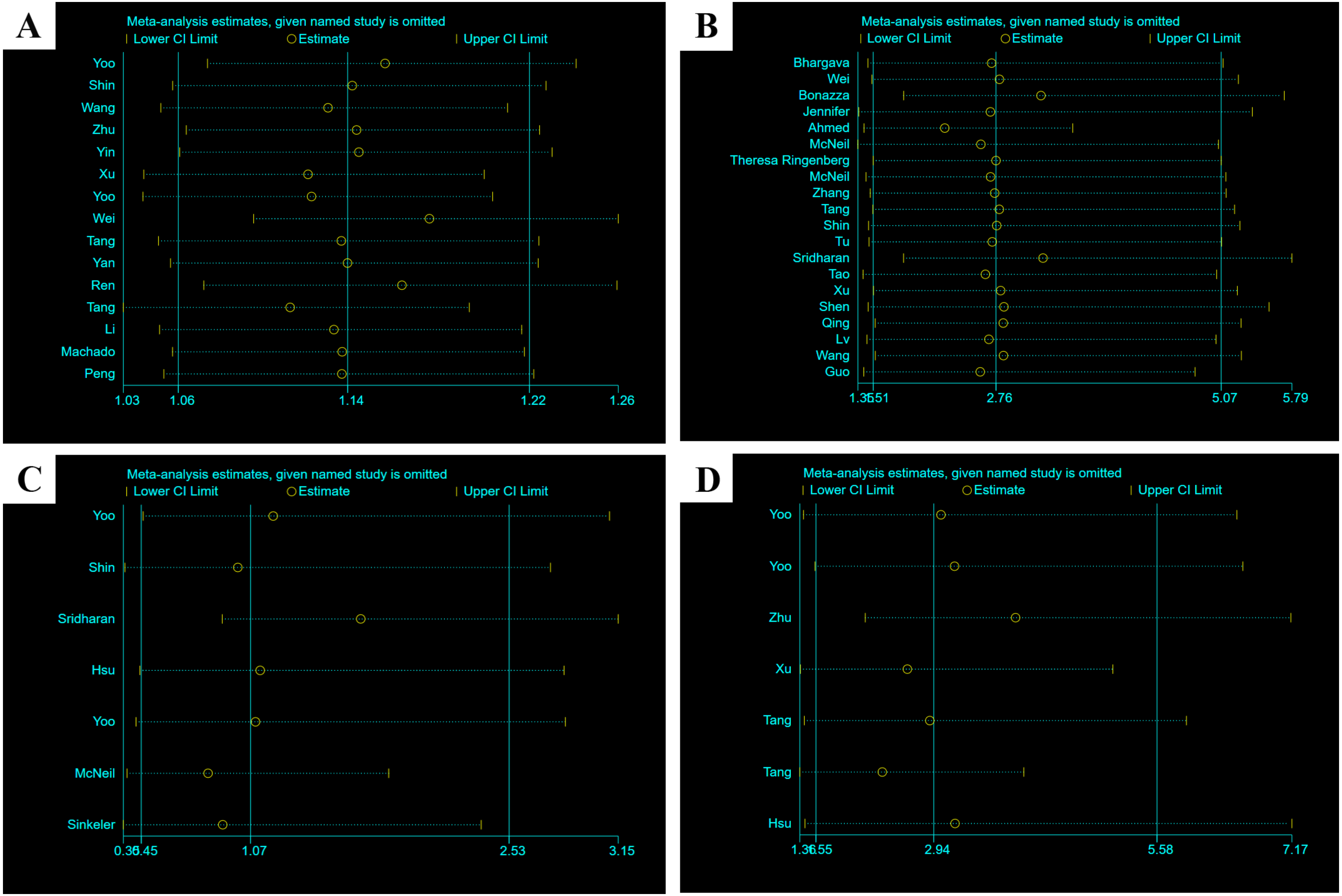

Fig.S1. Sensitivity analysis of clinical efficacy (A), nephrotoxicity (B), mortality (C), microbial clearance (D)
